# Molecular Structure, Membrane Interactions, and Toxicity of the Islet Amyloid Polypeptide in Type 2 Diabetes Mellitus

**DOI:** 10.1155/2016/5639875

**Published:** 2015-11-09

**Authors:** Lucie Caillon, Anais R. F. Hoffmann, Alexandra Botz, Lucie Khemtemourian

**Affiliations:** ^1^Sorbonne Universités, UPMC Univ Paris 06, Laboratoire des Biomolécules, 4 Place Jussieu, 75005 Paris, France; ^2^Département de Chimie, Ecole Normale Supérieure, PSL Research University, 24 Rue Lhomond, 75005 Paris, France; ^3^CNRS, UMR 7203 Laboratoire des Biomolécules, 75005 Paris, France

## Abstract

Human islet amyloid polypeptide (hIAPP) is the major component of the amyloid deposits found in the pancreatic islets of patients with type 2 diabetes mellitus (T2DM). Mature hIAPP, a 37-aa peptide, is natively unfolded in its monomeric state but forms islet amyloid in T2DM. In common with other misfolded and aggregated proteins, amyloid formation involves aggregation of monomers of hIAPP into oligomers, fibrils, and ultimately mature amyloid deposits. hIAPP is coproduced and stored with insulin by the pancreatic islet *β*-cells and is released in response to the stimuli that lead to insulin secretion. Accumulating evidence suggests that hIAPP amyloid deposits that accompany T2DM are not just an insignificant phenomenon derived from the disease progression but that hIAPP aggregation induces processes that impair the functionality and the viability of *β*-cells. In this review, we particularly focus on hIAPP structure, hIAPP aggregation, and hIAPP-membrane interactions. We will also discuss recent findings on the mechanism of hIAPP-membrane damage and on hIAPP-induced cell death. Finally, the development of successful antiamyloidogenic agents that prevent hIAPP fibril formation will be examined.

## 1. Introduction

Type 2 diabetes mellitus (T2DM) is classified as a protein-misfolding disease and shares the debilitating consequences of misfolded and aggregated peptides and proteins with more than 20 other diseases, such as Alzheimer's disease, Parkinson's disease, and spongiform encephalopathy [1–3]. T2DM is characterized metabolically by defects in both insulin secretion and insulin action, resulting in hyperglycemia, and is histopathologically characterized by the presence of fibrillar amyloid deposits in the pancreatic islets of Langerhans (islet amyloid) [4]. Amyloid is a generic term for a protein aggregation state in which the proteins bind to each other in a *β*-sheet conformation [5, 6]. In T2DM, amyloid deposits were initially assumed to be composed of insulin; however, in 1987 two different groups discovered that the major component of islet amyloid is a 37-residue polypeptide pancreatic hormone [7, 8], initially named insulinoma amyloid peptide [9], then diabetes-associated peptide [7], and finally islet amyloid polypeptide (IAPP) [8] or amylin [10]. The presence of these amyloid deposits in T2DM has been linked to the death of the insulin producing islet *β*-cells, thereby contributing to the development of this disease [4].

IAPP, found in all mammals, is coproduced and cosecreted with insulin in a molar IAPP : insulin ratio of 1 : 100 in healthy individuals, a ratio that can increase to 1 : 20 in T2DM. The function of hIAPP is still not entirely clear. As a paracrine hormone, hIAPP may be involved in the regulation of glucose metabolism, the control gastric emptying, the suppression of glucagon, the control of satiety, and other cellular processes [11–16]. Along with these functions hIAPP disrupts cell coupling and is also reported to induce apoptosis in isolated human islets [17]. Although hIAPP is a hormone, no specific receptors have yet been found. However, specific binding sites have been identified in the brain and in the renal cortex [18–20].

**Figure 1:**
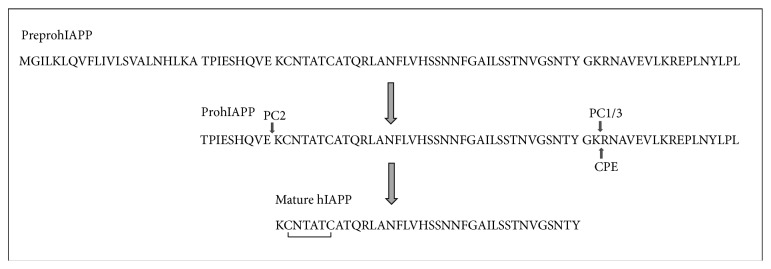
Processing of human PreproIAPP that lead to the formation of mature hIAPP. The cleavage site for PC2 and PC1/3 is indicated by arrows. The residues KR, indicated by arrow, which remain after the cleavage is induced by PC1/3 are removed by the carboxypeptidase E. This results in the amidation of the C-terminus of mature hIAPP. The disulfide bridge is shown on the mature hIAPP.

hIAPP is stored with insulin by the pancreatic islet *β*-cells and is released in response to the stimuli that lead to insulin secretion [21–23]. hIAPP is initially expressed by *β*-cells as an 89-aa residue preprohormone containing a 22-aa signal sequence which is cleaved off upon translocation across the endoplasmic reticulum, resulting in the prohormone precursor prohIAPP (Figure 1). Further processing of the prohormone proIAPP (67-aa in humans) involves cleavage at the C-terminal end either in the trans-Golgi network or in secretory granules, resulting in an intermediate 48-aa residue peptide. The second cleavage, at the N-terminal end, generates the mature 37-aa peptide, hIAPP, in the secretory granules. The two flanking peptides from prohIAPP remain in the secretory granules. Cleavage is initiated at two conserved dibasic sites and involves the two endoproteases prohormone convertase 2 (PC2) and prohormone convertase 1/3 (PC1/3) and the carboxypeptidase E (CPE), which are the same enzymes that process proinsulin to mature insulin [24–27]. A glycine residue at the start of the C-terminal propeptide acts as an amidation donor. The mature peptide undergoes posttranslational modification via formation of a disulfide bond between cysteine residues 2 and 7 (Figure 1).

There is a large and growing body of work on the biophysics of hIAPP amyloid formation and on the biological consequences of islet amyloid deposition. In this review, the current knowledge of hIAPP structure, hIAPP-membrane interactions, hIAPP toxicity, and the development of inhibitors of hIAPP toxicity will be presented and analysed.

## 2. Conformation and Structure of IAPP in Solution

hIAPP can appear in various states (monomer, oligomer, or fibril) all with very different structures. In solution, it has been shown, using circular dichroism, that monomeric hIAPP is a natively unfolded peptide which is predominantly random coil, aside from a rigid ring structure formed by the disulfide bridge between Cys2 and Cys7 residues. As for all amyloid forming peptides, hIAPP undergoes a conformational transition from its nonfolded state to a *β*-sheet structure, which increases over time [28–30]. This initial peptide conformational change is the key step leading to the formation of oligomers to highly ordered and insoluble amyloid fibrils.

Little information on the structure of hIAPP oligomers (and other oligomers associated with amyloid diseases) is available, mainly due to the instability of the species and to the relatively fast aggregation process of hIAPP. Both *β*-sheet-rich hIAPP oligomers and *α*-helix-rich hIAPP oligomers have been observed [31, 32]. High resolution microscopy (electron and atomic-force) and spectroscopy techniques (NMR) are most often used to detect oligomeric species, although NMR generally lacks the time resolution necessary to obtain a snapshot of oligomers. A handful of microscopy studies have confirmed that hIAPP oligomers consist of 10–20 hIAPP monomers with large variations in size and shape [33–35]. However, data on the size of hIAPP oligomers is somewhat scarce, where one study showed a range of 25–500 monomers and another showed a range of 20–40 monomers [36, 37].

The structure of hIAPP fibrils is more comprehensively described, probably due to the stable nature of the fibrils. Observation by electron microscopy (EM) of hIAPP fibrils reveals a polymorphism among the fibrils. In some cases, they organise themselves as helical fibrils of variable width, presenting some periodical twists. In other cases, the oligomers, also called “protofibrils” at this point, associate themselves laterally in long and striated ribbon-like strands. These strands, whose structure will be made more explicit later on, can be several nanometers long and have a width ranging from 5 to 15 nm [6, 38]. Further observation into the atomic organisation of these ribbon-like fibrils finds that the mature amyloid fibrils are characterized by a cross *β* structure, where all *β* strands, linked by interstrand hydrogen bonds, are oriented perpendicularly to the fibril axis. The insoluble and noncrystalline nature of hIAPP fibrils has complicated the determination of their molecular structure; however, further investigations using different techniques such as solid-state NMR spectroscopy or X-ray crystallography have provided two similar atomic level models for hIAPP fibrils. The first model was obtained using solid-state NMR spectroscopy in association with molecular modelling. The resulting model suggests that a single protofibril is made of two symmetric hIAPP monomers. The backbone of those hIAPP monomers possesses two *β*-strand segments formed by residues 8–17 and 29–37 separated by a bend or loop that is formed by residues 18–27. As the monomer structures itself into this hairpin, different orientations of the side chains of the residues between the two *β*-sheets have been obtained by Langevin dynamics. Either side chains of Gln10, Leu12, Asn14, and Leu16 are in contact with the *β*-sheet formed by residues 28–37, whereas side chains of Arg11, Ala13, and Phe15 are located on the outside of the fibril, or the organisation of the side chains is reversed, meaning that side chains of Gln10, Leu12, Asn14, and Leu16 are located outside the protofibril when side chains of Arg11, Ala13, and Phe15 are facing the core of the block. Each single monomer then interacts with another, as a pair, via the side chains of residues 26 to 32, thus forming the single protofilament. Protofilaments then laterally associate, leading to the mature fibril [6]. The second model for hIAPP fibrils was obtained by using X-ray crystallography and is based on steric zippers and on crystal structures that were obtained on segments 20–27 (NNFGAIL) and 29–33 (SSTNVG) of the peptide. This model, similar to that obtained by solid-state NMR with the exception of atomic distances between *β*-sheet layers, suggests that a monomer of hIAPP has a hairpin structure consisting of two *β*-strands. Each monomer then associates with another, with the SSTNVG segment of the first molecule creating a steric zipper that interacts with the NNFGAIL segment of the second. These stacks of peptides then associate themselves one on top of another, perpendicular to the fibril axis, to form the mature amyloid fibril [39].

## 3. Structure of Membrane-Bound hIAPP

**Figure 2:**
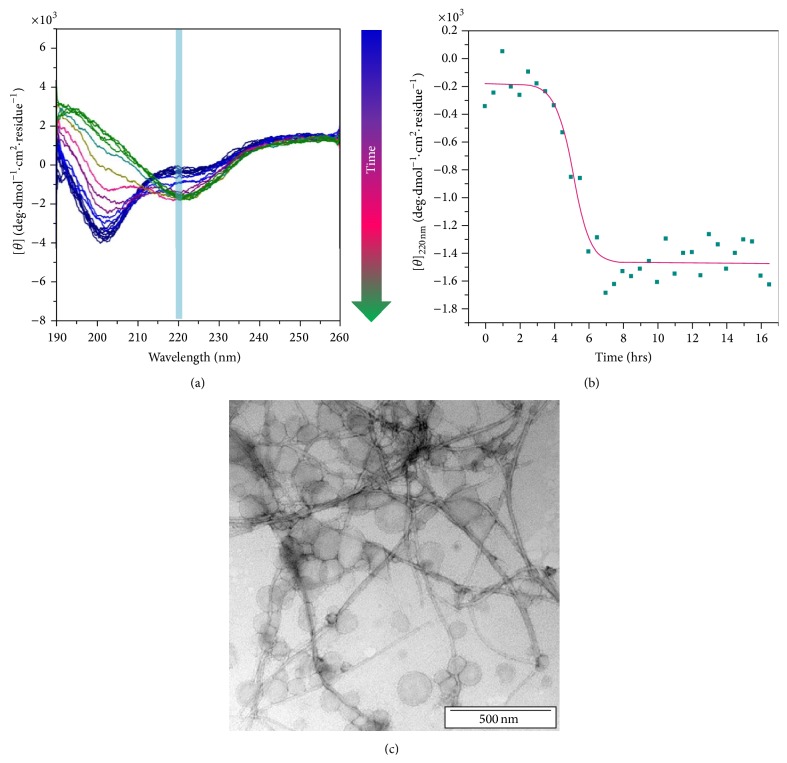
(a) CD kinetic study of hIAPP in vesicles. Plot color code: dark blue: CD spectrum recorded after 5 minutes and green: CD spectrum recorded after few hours. (b) Time course of CD ellipticity at 220 nm. (c) Negatively stained microscopy images of hIAPP after incubation with vesicles.

Aggregation of hIAPP on the membrane proceeds through a different pathway than in solution, as the structure of membrane-bound hIAPP is different to that of hIAPP in solution. The conformation of hIAPP has been examined using CD and NMR spectroscopy in different membrane models. In the presence of negatively charged membranes, hIAPP initially displays *α*-helical structure [40]. After a few minutes of incubation, the conformation of hIAPP changes to *β*-sheet, characteristic of fibril formation. hIAPP freshly added to zwitterionic membrane models (including among others phosphatidylcholine, phosphatidylethanolamine, cholesterol, or sphingomyelin) displays typical random coil conformation, which undergoes a typical change to *β*-sheet secondary structure in a few hours (Figure 2) [41]. In both anionic and zwitterionic micelles, the *α*-helical structure is predominant for several days, suggesting that in these media the peptide is kept in a monomeric conformation [41]. The micelle models enabled two groups to characterize the conformation of monomeric hIAPP, in SDS or DPC micelles using NMR [42, 43]. Both groups have found that the core (residues 7 to 28) is an *α*-helix structure with a kink region near residues 18–22. However, the presence of this kink is likely due to the high curvature of the micelles. The C-terminal part of hIAPP is unfolded with a high degree of flexibility, while the N-terminal part (residues 1–7) forms a hairpin due to the presence of the disulfide bond. The structure of hIAPP in the presence of membranes was also studied using microscopy techniques [44]. This study showed that hIAPP forms ion-channel-like structures in reconstituted membranes suggesting that these oligomeric hIAPP pores could insert in membranes and therefore change their barrier properties.

## 4. Mechanism of hIAPP Fibril Formation

**Figure 3:**
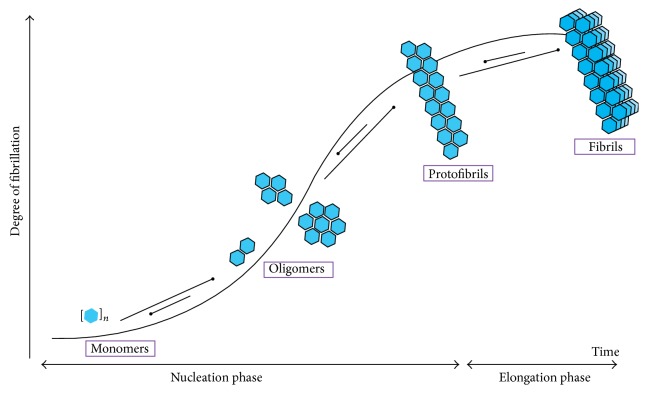
Schematic representation of fibrillation of hIAPP over time. During nucleation phase, hIAPP monomers associate themselves in order to form oligomers of various sizes. As the nucleation phase extends to the elongation phase, we can observe the formation of protofibrils, building blocks of the mature fibrils that characterize amyloidoses.

As for all amyloid peptides, hIAPP is produced as a soluble monomer and undergoes oligomerization and amyloid fibril formation via a nucleation-dependent polymerization process [45]. This process is divided into three main steps, in the first step, also named the lag phase, the peptide is in a monomeric form and/or in small soluble oligomers and no fibrils are present; the second step, called the elongation phase, is indicated by the propagation of the fibril growth with consumption of monomer and finally the plateau is reached when the amount of fibril remains constant. The kinetics of hIAPP fibril formation can be monitored in time by the commonly used method of specific binding of the fluorescent molecule Thioflavin T (ThT) to amyloid fibrils [46]. A kinetic trace of hIAPP fibril formation shows a lag phase and a sigmoidal transition which are both typical for fibril growth of amyloidogenic proteins and peptides (Figure 3). The lag phase is dependent on experimental conditions such as the peptide concentration, the ionic strength, the temperature, and the pH [47, 48].

**Figure 4:**
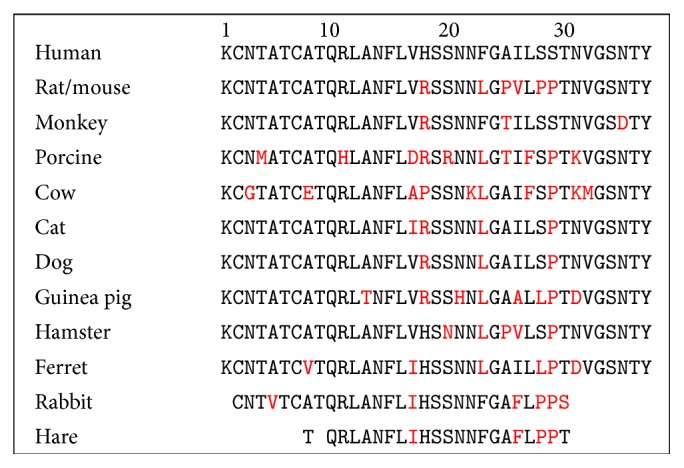
Primary sequence of IAPP from different species. Only partial sequences are available for rabbit and hare. Residues that differ from the human IAPP sequences are highlighted in red.

In most species, IAPP is expressed as an immature 89-membered amino acid peptide which is ultimately processed into a mature peptide of 37 amino acid residues [49]. Most of the N- and C-terminal residues, the intramolecular disulfide bridge, and the amidated C-terminus are strongly conserved throughout the mammalian species (Figure 4). There is a correlation between the sequence of IAPP and its propensity to form amyloid fibrils. For example, rat or mouse IAPP (rIAPP or mIAPP) differ from human IAPP by only six residues out of 37 and do not form fibrils. Note that those five of six positions between hIAPP and the nonamyloidogenic mIAPP are located between residues 20 and 29, the region which is known to be important in hIAPP fibrillation [50] and that three of the six residues involve a proline (at positions 25, 28, and 29) which is well-known as a disrupter of secondary structure and acts as a *β*-sheet breaker. Unlike rodents, dogs, and cow that do not form fibrils, primates, cats, porcine, ferret, and guinea pigs can form amyloid fibrils and are prone to T2DM [51].

Several studies have shown that hIAPP sequence can be divided into three parts, (i) the 1–19 region which is responsible for hIAPP/membrane interaction and insertion [52, 53], (ii) the 20–29 region, which is essential for amyloid fibril formation [29, 50], and (iii) the amyloidogenic 30–37 region which favours fibrils formation [54–56]. The N-terminal region contains all charged residues: Lys1, Arg11, and His18 [56]. In particular, the protonation state of the His18 is affected by the change in pH between the *β*-cell granules of the pancreas where hIAPP is stored at a pH of approximately 5.5 and released into the extracellular compartment, which has a pH of 7.4. Studies in solution have shown that hIAPP aggregation is faster at a pH of 8.8 than at 4.0 and that the fibril morphology is affected by a pH of 2.4 [56, 57], indicating that in solution the pH really plays a role in hIAPP aggregation. hIAPP contains one aromatic residue in each of the three main parts (Phe15, Phe23, and Tyr37), that raise the question of the importance of aromatic-aromatic and aromatic-hydrophobic interactions in IAPP aggregation. Studies using single, double, and triple mutants in which the aromatic residues were replaced by Leu residues (F15L, F23L, and Y37L) indicated that aromatic residues are not required for fibril formation. However, the substitution decreases the rate of fibril formation and alters the tendency of fibrils to aggregate [58–60]. The 20–29 region is the segment in which most mutations occur between the species (*vide supra*). Many substitutions that impact amyloid formation fall within the 20–29 domain confirming the importance of this region. A mutation (Ser → Gly) at position 20, which is found at low levels in certain Asian populations, was found to affect amyloid fibril formation and the development of T2DM* in vivo*. Indeed, this mutation seems to constitute a risk factor for diabetes, and it has been shown to increase the fibril formation rate* in vitro* [11, 61]. The effect of the proline residue has been further investigated on an 8–37 fragment of hIAPP, known to be amyloidogenic [62], but presenting substitutions by prolines in positions 17, 19, and 30. This study has shown that proline substitution outside the core 20–29 region of hIAPP not only reduces the aggregation of hIAPP in solution but also induces instability in the *β*-sheet structure. It is therefore suggested that proline substitution has a dominant negative role in fibril formation by either disruption of the nucleation process of hIAPP or by favouring the nonstructured state of the peptide [63]. A ≪reverse study≫ has been performed by Green and coworkers on rIAPP [64]. In this case, the proline residues have been conserved while Arg18, Leu23 and Val26 have been substituted by His18, Phe23, and Ile26 as in hIAPP. Results have shown that although the modified rIAPP does not complete the fibril formation to its maturity as would the wild type hIAPP, the peptide is able to form fibrils. This implies that the presence of the prolines in rIAPP does disrupt fibril formation but is not completely sufficient to avoid it. Moreover, these different studies also show the importance of key residues in hIAPP that influence its structure and induce mature fibril formation.

The mechanism of islet amyloid formation is not well understood. One potential cause has been proposed to be alterations in the processing of the hIAPP precursor molecule, prohIAPP, by the islet *β*-cells [26, 65]. Recent investigations have demonstrated that the precursor does not form amyloid aggregates in solution and may be important in early intracellular amyloid formation [27, 66, 67]. For example, several studies demonstrated that proIAPP interacts with heparin sulphate proteoglycan of the basement membranes that may act as a seed for amyloid formation [68]. In addition, it was shown that incomplete processing has large consequences for the properties of hIAPP and that these consequences point toward a less cytotoxic activity of the precursor as compared to mature hIAPP [69].

Another characteristic of hIAPP is the intramolecular disulfide bridge between Cys2 and Cys7 at its N-terminal, which was shown to be essential for its biological activity [70].* In vitro* studies highlight that the disulfide bond is not involved in the amyloid fibril core structure, prohibiting the N-terminal region of hIAPP from forming *β*-sheet structures. However, it does contribute to the assembly mechanism since the loss of the disulfide bond reduces fibril formation [62].

## 5. The Role of Lipid Membranes in hIAPP Aggregation

Membranes are implicated in hIAPP aggregation, both as the target of toxicity and as a catalyst [32, 71, 72]. hIAPP is known to interact with the membranes and to be inserted into the membranes, which affect hIAPP aggregation [52, 73]. The analysis of the first step of hIAPP/membrane interaction shows that hIAPP is inserted into phospholipids membranes most likely as a monomer and that the N-terminal part (1–19) is responsible for insertion [49]. This is in agreement with theoretical predictions from the amino acid sequence which suggest that only the 1–8 region has a membrane-interacting ability [74]. A study found that the disulfide bridge located in the N-terminal part (1–19) has a minor effect on membrane insertion properties and peptide conformational behaviour, suggesting that this disulfide bridge does not play a significant role in hIAPP/membrane interactions [75].

It is known that lipid membranes can promote hIAPP aggregation [71]. Lipid composition is a key factor that governs the extent to which membranes alter peptide aggregation. Several compositions were studied, highlighting the influence of various lipids on hIAPP aggregation and fibrillation. It has been shown that anionic lipids such as phosphatidylserine (PS) and phosphatidylglycerol (PG) strongly accelerate the kinetics of fibrils formation, thus reducing the lag time of the kinetics [40, 41, 71, 76]. In the presence of such membranes, hIAPP fibril formation occurs within a few minutes as opposed to a few hours in their absence. In contrast, the zwitterionic phosphatidylethanolamine (PE) is prone to slowing down these kinetics [41, 77]. Literature data thus indicate that the lipid composition of membranes has a large effect on hIAPP fibril formation kinetics but does not affect fibril morphology.

The modifications of fibril formation kinetics by lipid membrane composition could be attributed to peptide/lipid interactions. In particular, electrostatic interactions between anionic lipids and the positively charged hIAPP could explain the enhancement of hIAPP aggregation. Thus, as in solution, changes of pH, as well as ionic strength, could affect hIAPP aggregation and fibrillation in the presence of membranes [47, 48]. It has been shown that in the presence of membranes, a low pH decreases the rate of fibril formation, suggesting that a low pH prevents aggregation of hIAPP as well as membrane damage in the secretory granules [48]. The ionization state of the histidine residue significantly affected the kinetics of hIAPP conformational changes and concomitant fibril formation and this is directly related to the kinetics of hIAPP-membrane damage. These results confirmed that the change of protonation of His18 is very important in the kinetics of hIAPP aggregation and fibril formation.

Despite considerable progress in the field of hIAPP-membrane interaction, the mechanism of peptide-lipid interactions and membrane permeabilization still remains to be elucidated and it is not known how hIAPP-membranes interactions are related to cytotoxicity in T2DM.

## 6. hIAPP-Induced Membrane Damage

**Figure 5:**
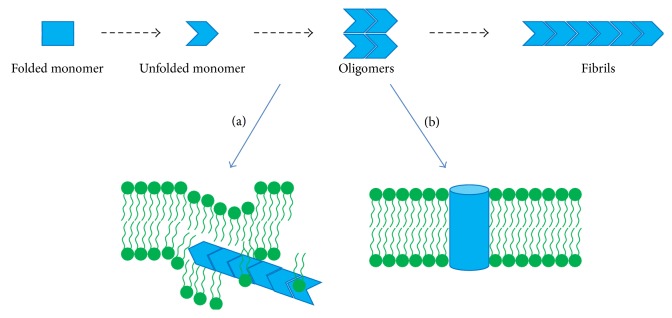
Schematic representation of permeabilization hypothesis. The natively fold peptide first starts to unfold. The first hypothesis (a) proposes that the monomeric peptide or small oligomers interact with the membranes and insert into the membranes. Fibril formation leads to membrane permeabilization by changes in membrane curvature and lipids recruitment. The second hypothesis (b) suggests that oligomeric species are toxic for the membrane interacting with it and forming pores.

The most widely accepted hypothesis is that hIAPP-induced cytotoxicity occurs via a membrane disruption mechanism (Figure 5). The first experimental evidence that an amyloid protein could cause membrane damage came from the work of Pollard [78]. It was found that the peptide A*β*, involved in Alzheimer's disease, could form cation-selective channels in planar lipid bilayers. A few years later, similar experiments were done on hIAPP and showed that hIAPP could also form cation-selective channels and ultimately disrupt the membranes [79]. On the other hand, neither the nonamyloidogenic mouse IAPP nor the amyloid hIAPP fibrils formed channels. These ion-channels have been also observed for other amyloidogenic proteins suggesting that the toxicity of amyloid proteins seems to be linked to their shared potential to form channels (or pores) in membrane [80, 81]. At this stage, it was clear that hIAPP could induce membrane damage; however, the exact mechanism of hIAPP-induced membrane disruption is far from clear and numerous models have been described during the last 15 years [33, 34, 36, 37, 52, 71, 79, 82–84]. A report concluded that soluble oligomers from several types of amyloids, including hIAPP, specifically increase lipid bilayer conductance, while fibrils and soluble low molecular weight species have no effect, suggesting that this may represent the common primary mechanism of pathogenesis in amyloid-related diseases [82]. It was also suggested that antimicrobial and amyloid peptides may share membrane-permeabilization mechanisms since these peptides share many characteristics. Indeed, for both peptides, a threshold peptide concentration is required to induce the oligomerization on the membrane surface which leads to the membrane damage. Recent studies on hIAPP and A*β* suggested that the amyloid fibril formation on the membrane surface induces membrane damage [84–86]. It was postulated that it is the growth of hIAPP fibrils at the membrane surface rather than the formation of oligomeric species that causes hIAPP-induced membrane damage. Thus, as soon as the fibril develops on the membrane surface, the structural integrity of the membrane is compromised, possibly by forcing the curvature of the bilayer to an unfavourable angle or by uptake of lipids by hIAPP fibrils during fibril elongation at the membrane (Figure 4). Uptake of membrane phospholipids in amyloid that forms at the membrane, as observed from* in vitro *studies [72, 76], as well as* in vivo *studies [87], could indeed be an additional factor that contributes to membrane leakage. Coarse-grained molecular dynamics simulation results agree with this hypothesis and showed that amyloidogenic peptides, including hIAPP, fibrillate on the surface of the membrane, damaging the vesicles and promoting leakage [88]. In all of these hypotheses, the membranes have an important role as mediator or accelerator of the conversion of one hIAPP species to the other. However, membrane disruption by hIAPP is not the only mechanistic hypothesis that has been proposed regarding *β*-cell death linked to the presence of the peptide; other mechanisms will be discussed next.

## 7. hIAPP-Induced Cell Toxicity

A primary question resides in the main location of hIAPP in the islet of Langerhans. As it has been described that amyloid deposits that are involved in T2DM appear to be extracellular, some evidence has suggested that the amyloid formation actually starts intercellularly. Indeed, several studies, performed either on transgenic mice capable of secretion of hIAPP or on baboons, have reported that hIAPP fibrils or in prefibrillar states could be observed either freely in the intracellular medium, locating the development site of the peptide's oligomers in the endoplasmic reticulum (ER), Golgi, or secretory granules of the *β*-cells [27]. Localisation of fibrillar species intracellularly may be particularly important as it could be the root of extracellular deposition of amyloid fibrils on pancreatic *β*-cells and imply different mechanisms of cellular death. Since the presence and oligomerization of hIAPP is related to dysfunction followed by apoptosis of pancreatic *β*-cells, different cell factors have been investigated in order to determine the origin and mechanism of the decrease of *β*-cell mass in the pancreas. As the source of amyloid formation is the misfolding of a specific peptide, studies have focused on the likely correlation between hIAPP synthesis and ER stress.

The ER serves many different functions in the cell, including assuring the correct native folding and posttranslational modification of peptides and proteins synthesized within the cell but also transportation of those molecules to the Golgi and secretory granules and release into the extracellular matrix. Those properties of the ER are well-balanced and regulated to avoid any misfolding and aggregation of proteins or peptides. However, this equilibrium can be disrupted by any ill-factors such as disturbances in redox regulation or calcium regulation and viral infection, applied on the ER. In particular, and as previously stated, insulin resistance results in a higher biosynthesis of insulin and thus of hIAPP. The consequent overproduction of protein and peptide in the *β*-cells then results in ER stress and triggers some malfunction in the folding process of the molecules, as it reaches overcapacity. The accumulation of misfolded protein in the cells along with ER stress cascades into the unfolded protein response (UPR). This regulation process involves simultaneously the production of chaperones to both assist the folding of proteins and limit their aggregation; reducing ER workload by inhibiting the protein synthesis triggering the UPR; enhancing the transportation of misfolded protein to the ubiquitin-proteasome system for degradation; and, as a last resort, triggering of the apoptosis process.

In spite of the various regulation responses to counteract the misfolding of proteins or peptides following ER stress, it is observed that hIAPP still autoassociates and forms toxic oligomers. This behaviour suggests that the prevention mechanism against hIAPP misfolding and therefore aggregation can be saturated and rendered noneffective. Different hypotheses regarding this fact can be evoked among which is the decrease in *β*-cell mass, also linked to apoptosis, enhancing once more the joint synthesis of insulin and hIAPP or the inability of the cell to eliminate cytotoxic oligomers once they are formed in the system.

To a larger extent, whether the cells are exposed to high concentration of hIAPP and/or if the responses to the peptide's aggregation are revealed to be inefficient, *β*-cell apoptosis is observed. Although mechanisms of the apoptotic behaviour of *β*-cells have yet to be fully elucidated, there have been some hypotheses that have been proposed concerning the different pathways and triggers that induce cell death. The first pathway, called the extrinsic pathway, involves extracellular factors such as membrane disruption, as described previously, or the binding to cell receptors. In particular, it has been described that exogenous or endogenous hIAPP could interact and thus activate the FAS receptor, present on the surface of cells. The activation of this “death receptor” results in apoptosis by in turn activating specific proteins such as caspase-3 [89]. The second pathway that has been described is the exogenous pathway and is linked to intracellular factors. Besides ER stress and UPR, mentioned before and mainly involved in pancreatic *β*-cells death, other factors disturbing the main function of ER are likely to enhance hIAPP oligomerization and cell death. Among those, mitochondrial dysfunction, generation of oxygen free radical, defects in autophagy can also be mentioned [90].

Lastly, it has also been suggested that *β*-cell toxicity can be induced by an inflammatory response linked to hIAPP. Indeed, it has been found that the insulin resistance and production of hIAPP initiate an increase in the concentration of proinflammatory cytokines such as interleukin 1*β* (IL-1*β*), which has been previously described to be cytotoxic to pancreatic islets of Langerhans [11, 27, 90–97].

## 8. Inhibition of hIAPP Fibril Formation

The amyloid pathway leading to fibrils is supposed to be responsible for *β*-cell death and T2DM. The development of inhibitors of amyloid formation is therefore of considerable interest in treating patients suffering from T2DM. However, although hIAPP is extremely amyloidogenic, most research has focused on other amyloidogenic proteins like A*β* peptide or *α*-synuclein, involved in Alzheimer's and Parkinson's disease, respectively. Different classes of inhibitors of hIAPP amyloid formation have been identified and have been tested for their ability to reduce amyloid cytotoxicity, using either cells or* in vitro* model systems [98–104].

First of all, insulin is one of the most effective inhibitors of hIAPP amyloid formation [71, 105–111]. However, little is known about the mechanism of this inhibition process. Some studies have demonstrated that insulin interacts with the growing hIAPP fibril [106, 108]. Another study showed that the mechanism of inhibition of hIAPP fibril formation by insulin is related to strong binding of the insulin *β*-chain to hIAPP [112]. A recent molecular modelling study has shown that it involves a helix-helix interaction between the helical insulin and the N-terminal helix of hIAPP. The interaction between insulin and hIAPP may stabilize hIAPP in a nonamyloidogenic monomeric state [111].

Another valuable class of inhibitors are the polyphenols, which are thought to interact with amyloidogenic proteins via aromatic *π*-*π* interactions, although the precise mechanism is an issue still under debate [102, 113–116]. The molecule (−)-Epigallocatechin 3-Gallate (EGCG), a natural component of green tea, is of particular interest [117, 118]. Indeed, EGCG could have the ability to bind the unaggregated hIAPP, leading to the formation of noncytotoxic oligomers through another pathway. Nevertheless, the mechanism remains under some debate [114, 115]. In addition to its inhibitory activity, EGCG is one of the few molecules able to disaggregate preformed hIAPP amyloid fibrils in bulk solution [117, 118]. Effects are observed for a 2 : 1 hIAPP to EGCG ratio and even for a 5 : 1 IAPP to EGCG ratio [118]. On the contrary, a 1 : 1 hIAPP to EGCG ratio is necessary to increase the cell viability in the presence of EGCG. This molecule is then less effective in the presence of cell membranes than in solution [118]. Morin hydrate (2′,3,4′,5,7-pentahydroxyflavone) is a polyphenol as well, and more precisely a flavonoid. This molecule inhibits the amyloid formation of hIAPP, since the inhibition is effective from a 1 : 1 hIAPP to Morin hydrate ratio. The molecule acts in a ratio-dependent manner, because the effects on fibrils formation are even more pronounced than when the molecule is introduced in excess [119]. As with EGCG, Morin hydrate is able to disaggregate preexisting fibrils at a one to one ratio. Unfortunately, all not hydroxyflavones are inhibitors of hIAPP amyloid formation. For example, Myricetin is an inhibitor of A*β* amyloid formation but is totally ineffective against hIAPP at a one to one ratio. The number and position of hydroxyl groups may also play a role in the mechanism of inhibition. However, it has been demonstrated that Myricetin slows down hIAPP amyloid formation in a 10-fold excess, that is, at very high concentrations. Nevertheless, this molecule is effective* in vivo* and merits further consideration. Equally of interest is phenol red, a small aromatic polyphenol molecule, which elicits an effect on hIAPP fibril formation at a 4-fold excess of molecule* in vitro*. Its high efficiency in protecting pancreatic *β*-cells from the cytotoxic effect of hIAPP makes it a particularly attractive target molecule. In addition, phenol red is a nontoxic and noncarcinogenic compound, in contrast to many polycyclic aromatics [120]. Unfortunately, the mode of action of this class of molecules on the hIAPP amyloid formation is not known and no clear mechanism has yet been suggested. Their interest lies in their ability to not only inhibit the formation of amyloid fibrils but also disaggregate existing fibrils, protecting cells against hIAPP amyloid cytotoxicity.

A third class of molecules which are active against hIAPP fibrillation are molecular mimics. This particular strategy is based on molecular recognition thanks to similar molecular structure. For example, rat IAPP (rIAPP) whose sequence differs from hIAPP at only six positions is nonamyloidogenic* in vitro* or* in vivo*. rIAPP inhibits hIAPP amyloid formation in a dose-dependent manner. Even if the slowdown of the aggregation kinetics exists at 1 : 1 or 1 : 2 hIAPP to rIAPP ratios, the effect on fibril morphology and final quantity is only observed in a 5-fold or 10-fold excess of rIAPP [121]. As in the case of insulin, a mechanism involving interactions between helical N-terminal regions of the two peptides has been suggested. In addition, efficient inhibition of hIAPP amyloid formation has been demonstrated by the modified aromatic peptide fragment NFGAILSS in which phenylalanine was substituted with tyrosine (NYGAILSS) [33, 120]. Replacement of this aromatic amino acid leads to the formation of a nonamyloidogenic peptide, aromatic residues playing a role in accelerating the process of fibrillation. Unfortunately, this peptide proved to be cytotoxic toward *β*-cells and thus cannot be used as an inhibitor [120]. A study showed that Aib modified peptide induced a high inhibition effect on the full-length hIAPP [122]. More recently, another analogue of hIAPP was designed by N-methylation of the amide bonds at G24 and I26, called IAPP-Gl [123]. This molecular mimic is a nonamyloidogenic hIAPP analog that is able to associate with hIAPP and thus inhibits the process of fibrillation and cytotoxicity. hIAPP-Gl was found to be a remarkable inhibitor of hIAPP amyloid formation. In fact, a 1 : 1 hIAPP to hIAPP-Gl ratio is sufficient to completely inhibit amyloid formation. Moreover, hIAPP-Gl dissociates existing oligomers and fibrils and reverses their cytotoxicity [123].

Finally, an original compound, selenium phycocyanin (Se-PC), has been discovered as an inhibitor, acting in a dose-dependent manner [124]. The combination of Se and phycocyanin proved to be particularly effective at stopping the fibrillation process of hIAPP. In fact, Se-PC is effective at even a 4-fold less concentration relative to hIAPP. A mode of action has been proposed according to which Se-PC interferes with hIAPP to interrupt the fibrillation process thanks to the formation of nanoscale oligomers. This compound is a good inhibitor of the *β*-cell death induced by hIAPP. Se-PC is thus a promising candidate for antidiabetes drug development due to its activity on the cell media.

Unfortunately and despite considerable effort, the mechanism of hIAPP amyloid formation is not understood nor the mode of action of most of the hIAPP amyloid inhibitors. It is even more difficult to understand these mechanisms as most of the experiments described refer to studies in bulk solution [125].* In vitro* studies in diluted bulk solution do not adequately reflect the complexity of the cellular surrounding. Thus, the effect of inhibitors can be widely changed according to the medium. This is especially the case for the inhibitor EGCG whose inhibitory activity is lower than that in bulk solution [117]. AFM images confirm the presence of abundant fibrils at the phospholipid interface, even in a large excess of EGCG, whereas this molecule is very effective in bulk solution.

It is important to note that some publications referencing hIAPP inhibitors only draw conclusions from ThT assays. The monitoring of amyloid fibril formation via ThT experiments is a very convenient and common technique, but with a large disadvantage concerning the study of inhibitors. Indeed, many potential inhibitors can interfere with the ThT dye, thereby inhibiting the fluorescence of the probe and leading to false positive inhibitors. This is particularly the case for rifampicin or hydroxyflavones that interfere with ThT and might suggest that they inhibit hIAPP amyloid formation, which is contradicted by TEM images [119, 126]. It is thus necessary to check the results obtained by ThT fluorescence and to confirm the inhibitory activity with other techniques such as circular dichroism (CD), nuclear magnetic resonance (NMR), transmission electron microscopy (TEM), or atomic force microscopy (AFM).

## 9. Conclusions

Today, there are 382 million people living with diabetes. Diabetes is on the rise all over the world and medical practitioners are struggling to keep pace. Worldwide, one person dies as a consequence of diabetes (such as cardiovascular disease, kidney failure, and lower limb amputation) every 6 seconds. In this regard, there is currently great interest in the field of islet amyloid. However there are important outstanding issues. Important questions that remain to be answered include the following. What is the mechanism of hIAPP fibril formation* in vivo*? What are the morphology and structure of hIAPP oligomers and hIAPP fibrils* in vivo*? Why do oligomers and amyloidogenic protein form? What is the exact nature of the toxic species? Much of the research work on hIAPP-membrane structure and hIAPP-membrane interactions is performed on membrane models. Progresses have been made and the results from the biophysical studies have generated some hypotheses. However, an important challenge will be now to connect these biophysical results with the* in vivo* experiments.
